# The Future Is Big—and Small: Remote Sensing Enables Cross-Scale Comparisons of Microbiome Dynamics and Ecological Consequences

**DOI:** 10.1128/mSystems.01106-21

**Published:** 2021-11-02

**Authors:** Deanna S. Beatty, Lillian R. Aoki, Olivia J. Graham, Bo Yang

**Affiliations:** a Evolution and Ecology Department, University of California, Davis, California, USA; b Data Science Initiative, University of Oregon, Eugene, Oregon, USA; c Department of Ecology and Evolutionary Biology, Cornell University, Ithaca, New York, USA; d Department of Urban and Regional Planning, San Jose State University, San Jose, California, USA; Tufts University

**Keywords:** geographic information systems, machine learning, metabolomics, microbiome, modeling, remote sensing, spatial ecology, unmanned aerial vehicle

## Abstract

Coupling remote sensing with microbial omics-based approaches provides a promising new frontier for scientists to scale microbial interactions across space and time. These data-rich, interdisciplinary methods allow us to better understand interactions between microbial communities and their environments and, in turn, their impact on ecosystem structure and function. Here, we highlight current and novel examples of applying remote sensing, machine learning, spatial statistics, and omics data approaches to marine, aquatic, and terrestrial systems. We emphasize the importance of integrating biochemical and spatiotemporal environmental data to move toward a predictive framework of microbiome interactions and their ecosystem-level effects. Finally, we emphasize lessons learned from our collaborative research with recommendations to foster productive and interdisciplinary teamwork.

## PERSPECTIVE

Exploring Earth's microbial diversity provides a deeper understanding of microbial interactions that structure ecosystems and shape biodiversity (1, 2). For example, the discovery of the most abundant photosynthetic organism on Earth, *Prochlorococcus* (3), redefined our understanding of the microbial contribution to global primary productivity and marine trophic dynamics (4). Found in oligotrophic oceans, *Prochlorococcus* produces an estimated 4 gigatons of fixed carbon annually (4, 5). Similarly, in host-symbiont systems, microbial primary productivity fuels ecosystems by feeding habitat-forming hosts; corals that house intracellular microalgal symbionts and cover <1% of the ocean floor create habitat for ∼25% of marine biodiversity (6, 7). Use of microscopy and careful laboratory experiments led to some of the great microbial discoveries of the 20th century; omics-based molecular tools expand our capacity to study the ecology and evolution of microbes in the 21st century (8).

For (microbial) research, the future is big—and small. Scientists can leverage powerful, cross-disciplinary approaches that pair omics tools with remote sensing and spatial statistics to study spatiotemporal variation in microbiome interactions. For example, by studying *Vibrio*-plankton interactions, climate, and genomics, researchers understand how humans facilitate Vibrio cholerae dispersal and how rising sea surface temperatures and heavy rainfall promote Vibrio cholerae outbreaks (9–12). Leveraging remote sensing for monitoring and predicting pathogen outbreaks or spread in natural and agricultural systems, like tracking the emerging infectious pathogen *Phytophthora ramorum*, which causes sudden oak death, or detecting presymptomatic leaf stripe disease in grapevines (13–15), illustrates the power of creative, collaborative science. Here, we discuss the use of remote sensing and machine learning with omics-based technologies to create an atlas of host-associated and free-living microbes and their diverse ecologies. We highlight ecosystems where scaling microbial interactions through space and time may be particularly fruitful for understanding consequences for populations and communities.

## NEW FRONTIERS FOR REMOTE SENSING OF MICROBIAL DYNAMICS AND DYSBIOSIS

**FIG 1:**
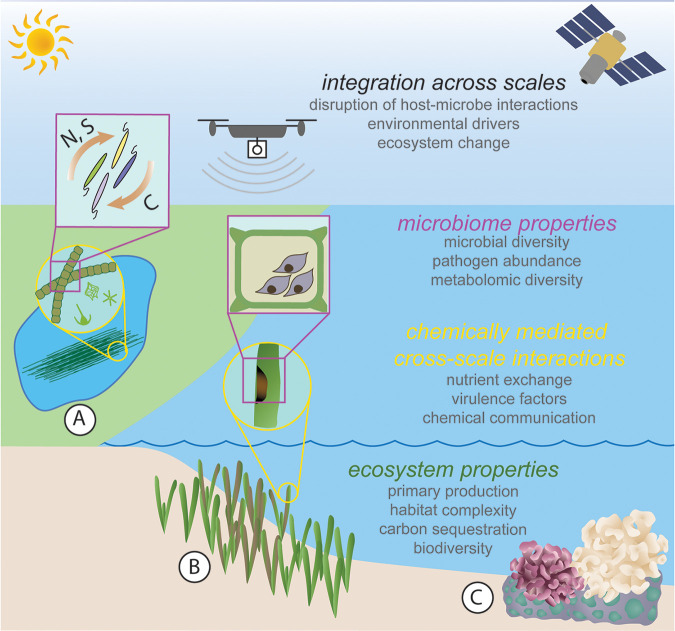
Remote sensing in aquatic and marine ecosystems can reveal how microbiome dynamics cascade across scales to impact ecosystem functions. (A) In freshwater ecosystems, cyanobacterial blooms impact drinking water and human and wildlife health (23); composition and persistence of these blooms may be linked to nutrient cycling by heterotrophic bacteria in the phycosphere of microalgae or cyanobacteria (22, 27). (B) In seagrass meadows, the infectious pathogen *Labyrinthula zosterae* causes seagrass wasting disease by invading plant tissue and attacking chloroplasts, with severe outbreaks causing shoot mortality and meadow decline (58); aerial images can detect changes in meadow extent (17) and may detect damage to seagrass tissue (18). (C) Intertidal and subtidal coral reefs are another system where remote sensing of microbial dynamics underpinning ecosystem disturbance (e.g., coral bleaching) may be possible as algorithms advance and hyperspectral sensors become more affordable (18, 21, 32). Figure created by Lillian R. Aoki, with feedback and contributions from all authors. Symbols provided courtesy of the Integration and Application Network (ian.umces.edu/media-library).

**FIG 2:**
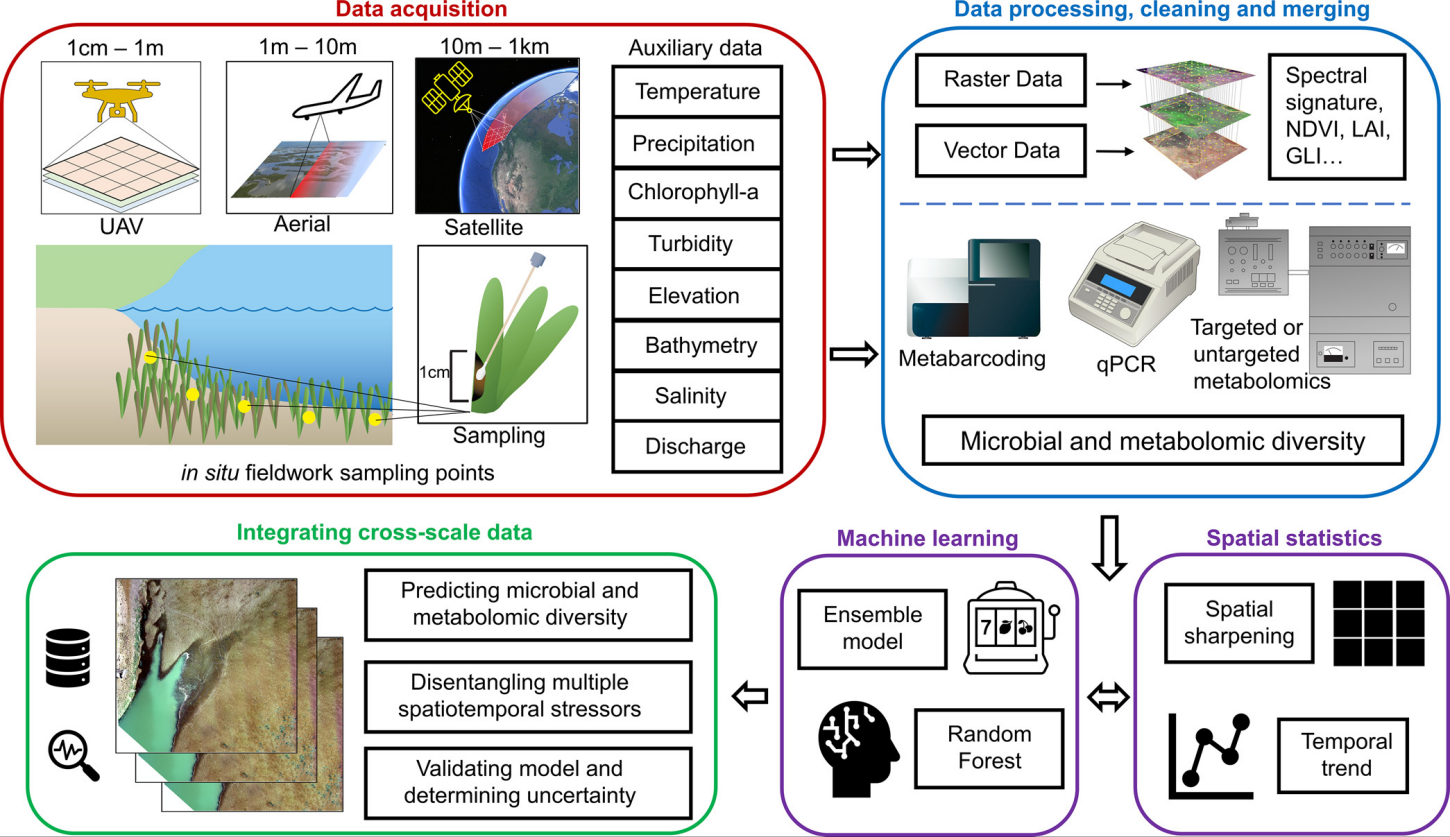
Overview of process linking remote sensing and microbial approaches. (A) Images collected from UAV, aerial, and satellite, coupled with auxiliary remotely sensed and publicly available data products, provide spectral and environmental site characteristics. Researchers collect *in situ* microbial samples in tandem with identifying macroorganism diversity and composition. (B) Use of a geographic information system (GIS) to process remote sensing data into raster data and paired auxiliary and *in situ* data into vector data with geographical coordinates. Derivation of spectral signature, normalized difference vegetation index (NDVI), leaf area index (LAI), green leaf index (GLI), and other metrics from remote sensing imagery and characterization of metabolomic and microbial diversity with established protocols (8, 42, 43) allow connections between remotely sensed ecosystem traits and microbial dynamics to be discerned. (C) Assimilation and fusion of multisource data with spatial statistics, including spatial sharpening and temporal prediction, increases coverage and resolution of data sets (44, 45). Random forest regression algorithms and ensemble methods predict microbial and metabolomic dynamics with remotely sensed and *in situ* data. (D) By coupling multisource data from remote sensing and environmental and *in situ* sampling, calibrated models generate environmental characteristics, predict microbial and metabolomic diversity, and disentangle multiple stressors with uncertainty estimates. Figure created by Bo Yang, with feedback and contributions from all authors. Symbols provided courtesy of the Integration and Application Network (ian.umces.edu/media-library) or reprinted from TogoTV (© 2016 DBCLS TogoTV) with permission.

Advances in remote sensing technologies provide new opportunities to understand microbial dynamics. Global-scale observations from satellites enable spatial resolution of 10 m to 1 km with a revisit cycle of days to weeks (16). More recently, unmanned aerial vehicles (UAVs) provide more flexible methods with up to 1 cm spatial resolution and on-demand mapping capabilities (17). UAVs can detect changes in plant microbiomes and phytoplankton communities through spectral signatures (Fig. 1) (13, 18, 19). These applications may be adapted to other habitat-forming species and their microbiomes, like those in grasslands, kelp forests (20), or coral reefs (21), as hyperspectral sensors become more affordable (18). Importantly, UAVs fly at low altitudes, circumventing cloud interference that can result in incomplete data sets from satellites. Thus, UAV imagery provides a novel avenue for correlating ecological observations with microbial dynamics over space and time. Rich environmental data sets from satellite remote sensing can supplement fine-scale UAV imagery (e.g., temperature, precipitation, and turbidity in Fig. 2). We present two case studies that illustrate how remote sensing—when coupled with omics approaches—can improve monitoring and prediction of macroscale phenomena.

## CASE STUDY: PHYTOPLANKTON BLOOMS

Combining microbiome and remote sensing methods can advance our understanding of phytoplankton bloom dynamics (Fig. 1). Harmful algal blooms (HABs) are of critical concern, as they impact human and wildlife health (22, 23). However, complex bloom dynamics—formation, composition, persistence, and toxicity—are challenging to predict (24, 25). Manual probe measurements are slow and provide limited coverage. In contrast, satellites, aerial flights, and on-demand UAVs can collect spectral imagery (chlorophyll-*a* and phycocyanin pigments) (Fig. 2) to quantify spatial and temporal bloom dynamics rapidly and across large areas (19, 26). These methods measure cyanobacteria or algal biomass and can evaluate potential environmental drivers (auxiliary remotely sensed data products) (Fig. 2) but cannot detect toxin presence or differentiate species within blooms.

Interactions within plankton communities and among heterotrophic bacteria and bloom-forming species (22, 27) indicate that omics approaches (e.g., 16S metabarcoding, metagenomics, and metabolomics) (Fig. 2) coupled with remotely sensed algal or cyanobacterial biomass (26) would be a powerful approach to predict bloom dynamics. For example, comparisons between microbial community composition, concentrations of the cyanobacterial toxin microcystin, and microcystin biosynthesis genes suggest that algicidal and microcystin-degrading bacteria may control toxicity of cyanobacterial blooms (28). With the advent of UAV imaging technology, biomass and high-resolution spatial measurements of cyanobacteria (19) can be paired with metabolomic surveys targeting toxins, allelopathic compounds (25), or vitamins and nutrients within bloom communities (22). Repeated measures at flexible and relevant temporal scales (e.g., days to months for bloom formation and persistence) and spatial scales (e.g., across salinity, temperature, or turbidity gradients) can reveal linkages between macroscale and microbial dynamics and environmental drivers. We can develop a predictive framework of toxic bloom formation and persistence by synthesizing these approaches.

## CASE STUDY: SEAGRASS DISEASE

Disease outbreaks in seagrass meadows present another promising case study for applying remote sensing to understand microbial dynamics. Seagrasses create habitat that supports biodiversity but are declining globally due to multiple stressors, including disease (29, 30). Meadows grow in shallow coastal waters; intertidal seagrass can be mapped at low tide by UAVs (17, 31), and water correction algorithms allow mapping of subtidal meadows (32, 33). We can derive ecosystem-level characteristics such as plant biomass and above-ground carbon stocks from UAVs and satellite measurements in response to disease outbreaks (34, 35). Further, UAVs (1-cm resolution) (Fig. 2) can detect foliar plant pathogens that alter leaf spectral signatures; these advances are currently implemented to manage pathogens in agricultural systems (13, 18) and have the potential to detect wasting disease lesions on seagrass leaves (Fig. 1).

Despite widespread occurrences of seagrass wasting disease outbreaks and vast consequences for marine ecosystems (29, 30), causes of disease are not well understood (30). Multiple stressors may interact to suppress plant immune function and/or photosynthesis and may also promote pathogen growth or virulence (29, 30, 36, 37). Remotely sensed data (Fig. 2) can be used to test for interactive effects of stressors, such as light-limiting algal blooms from chlorophyll-*a* and phycocyanin, warming from thermal sensors, and freshwater discharge events (13, 19, 26, 38) on disease outbreaks. Indeed, remotely sensed thermal anomalies were recently linked to wasting disease severity (L. R. Aoki, B. Rappazzo, D. S. Beatty, L. K. Domke, G. L. Eckert, M. E. Eisenlord, O. J. Graham, L. Harper, T. L. Hawthorne, M. Hessing-Lewis, K. Hovel, Z. L. Monteith, R. Mueller, A. M. Olson, C. Prentice, C. Ritter, J. J. Stachowicz, F. Tomas, B. Yang, J. E. Duffy, C. Gomes, and C. D. Harvell, submitted for publication). By collecting geospatially paired omics data such as root metabolomes, foliar microbiomes, or quantitative abundances (qPCR) of pathogens (Fig. 2), we can obtain a more holistic understanding of how stressors may interact to disrupt beneficial microbiomes (39) or promote pathogenic microbiomes (40). Thus, by coupling high-resolution UAV-based detection of foliar diseases and satellite-sensed biotic and abiotic stressors, we can move toward a synthetic understanding of the ecological consequences of microbial dysbiosis.

## APPLICATIONS FOR REMOTE SENSING, MACHINE LEARNING, SPATIAL STATISTICS, AND OMICS DATA

We can model, monitor, and predict ecological change by pairing UAV, aerial, and satellite remote sensing with *in situ* surveys. From sensors that detect visible (red, blue, and green), near-infrared, microwave, and thermal bands, we can derive variables such as chlorophyll-*a* levels, spectral signatures for green leaf index (GLI), normalized difference vegetation index (NDVI) for above-ground plant biomass, phycocyanin for cyanobacterial biomass, and temperature (13, 16, 18, 26). Discussions on sensors and derived products can be found in detail elsewhere (13, 16, 18, 41). Additional data from meteorological and hydrological stations and other publicly available data sets provide environmental site characteristics. Remote sensing data are processed into raster data (e.g., gridded pixel matrix in Fig. 2) within a geographic information system (GIS, such as ArcGIS or ENVI) and validated against *in situ* data using geographical coordinates (i.e., vector data) (Fig. 2). *In situ* sampling enables ground-truthing aerial images with microbial indicators from 16S metabarcoding, metagenomics, and targeted or untargeted metabolomics (Fig. 2) processed with established protocols (8, 42, 43). Further, spatial statistics can fuse remotely sensed data collected at varied spatial resolutions and temporal frequencies from satellites, planes, and UAVs for better coverage (44, 45). With machine learning and statistical modeling, implemented with training data and model validation, we can predict microbial dynamics and their ecological consequences. For example, georeferenced data sets may indicate microbial or chemical predictors of remotely sensed environmental change like algal blooms (19, 26), plant productivity or biomass (34, 35, 46), and disease (13, 14, 18). These approaches can be applied to aquatic, marine, and terrestrial ecosystems.

## LEVERAGING CROSS-SYSTEM COMPARISONS

Between-system comparisons reveal shared knowledge gaps in understanding how microbial interactions influence ecosystem-level change. Microbes underpin ecosystem function (2, 47), partially due to their diverse metabolisms and ability to respond rapidly to environmental perturbations, including global change stressors (48, 49). Microbiome metrics may be particularly suited to identify and predict large-scale shifts in ecosystem structure and function (46, 50). This requires integrating knowledge of how stressors impact hosts, microbes, and host-microbe interactions through time and space (51), a challenge for aquatic ecosystems, where chemical and microbial diversity—and their dynamics—remain underexplored (52).

A deeper understanding of host-microbe interactions can help identify microbial signs of ecosystem perturbations. For example, commensal nutrient cycling bacteria are critical to productivity of seagrasses (53), corals (54), and phytoplankton (22, 27); disruption of nutrient exchange between heterotrophic bacteria and primary producers is linked to coral bleaching (55), harmful algal blooms (56, 57) and seagrass disease (58). Yet the factors that disrupt nutrient cycling within these complex communities are not always clear, leaving the likelihood, timing, and spatiotemporal extent of large-scale ecosystem change uncertain. In seagrass rhizospheres, environmental stress can shift dominant methylotrophic, nitrogen-fixing, and iron-cycling bacteria to sulfur-cycling bacteria, but the consequences of these changes for seagrass and ecosystem health remain unclear (59). A better understanding of host-microbe chemical interactions—including nutrient, vitamin, and substrate exchange, growth and virulence factors, and predation and defense (27, 60)—is key to identifying appropriate microbial indicators of ecosystem change.

Synthesizing omics data with physiochemical environmental drivers can address important knowledge gaps. In some cases, microbial data can be early warning indicators, revealing ecosystem impacts like disease outbreaks or microbial dysbiosis prior to macroscale observations (40, 48, 50). For instance, under warming conditions, changes in coral mucus bacterial community and sugar composition can precede visible bleaching symptoms that occur when corals expel their photosymbionts (61). Understanding microbial change requires baseline data from a healthy or undisturbed state and consideration of multiple potential triggers, including factors such as host resistance and local and global environmental stressors (40, 48). New approaches can leverage machine learning to identify relevant predictors from microbial community data (46, 50) and apply spatial statistics to connect discrete *in situ* samples to continuous, remotely sensed data (62). Coupling these methods to develop predictive models can expand the spatiotemporal scales across which we understand ecosystem-level microbial impacts. Failure of predictive models can also indicate ecologically significant interactions that warrant further investigation (63).

The need for predictive ecological modeling is accelerating in tandem with global environmental change (64). Microbiomes are increasingly recognized as foundational to ecosystem services, underscoring the need to assess the influence of multiple stressors on microbial and ecosystem change (49). Synthesizing microbial and remotely sensed data through time and space is resource-intensive and interdisciplinary; collaborative networks are critical to illuminate the cascade of cross-scale interactions that lead to disease outbreaks, changes in biodiversity, and other shifts in ecosystem status and function.

## CONCLUSION

What can we learn from an interdisciplinary approach of integrating microbial, remote sensing, and machine learning methods? Coupling microbial and remote sensing data may allow more accurate predictions about how microbiomes may shift in future climate change scenarios (9, 11, 14, 56) and, in turn, better estimate the biogeochemical, ecosystem-level impacts of such microbial shifts, like carbon and nutrient cycling. We suggest that these approaches will enable more precise predictions of where and when harmful algal blooms (56) or disease outbreaks (13, 18) are likely to occur and their effects on carbon storage or losses across variable landscapes (34, 35). Such knowledge may improve conservation and management of natural resources by informing the timing and location of plant restoration activities, coastal development projects, or recreational use of land and waterways. Additionally, microbial indicators from omics data could inform adaptive management strategies to reduce disease incidence or severity. Albeit somewhat controversial, manipulating microbiomes with probiotics (65) or antibiotics (66) could reduce disease severity in corals and other foundation species. Remote sensing may be able to help identify and prioritize disease-susceptible populations for probiotic treatment (13–15). Because climate change rapidly creates novel environmental conditions, we urgently need better predictors of nonlinear ecosystem change to develop adaptive management plans.

Successful implementation of paired geospatial and omics research approaches will require collaboration among ecologists, microbiologists, computer scientists, and geographers. To push our fields forward, we need structured workshops to foster connections, develop skills, and infuse expertise and creativity into our shared research initiatives. For example, training workshops that bring together users of geospatial, machine learning, and omics tools to brainstorm unique data use, storage, and analyses are needed to create efficient data pipelines and promote novel findings. Importantly, teams should include and support individuals from historically excluded and marginalized groups, disparate research fields, and career stages. Doing so will create more equitable opportunities to engage in research (67). We advocate that teams also participate in training on best practice for working in large, interdisciplinary groups (68). By applying these approaches, together we can advance the field of microbiome research, connecting microbial interactions to dynamic ecosystem functions across time, space, and disciplines.
